# Cardiac Aging: From Basic Research to Therapeutics

**DOI:** 10.1155/2021/9570325

**Published:** 2021-03-09

**Authors:** Mingjing Yan, Shenghui Sun, Kun Xu, Xiuqing Huang, Lin Dou, Jing Pang, Weiqing Tang, Tao Shen, Jian Li

**Affiliations:** ^1^The Key Laboratory of Geriatrics, Beijing Institute of Geriatrics, Beijing Hospital, National Center of Gerontology, National Health Commission, Institute of Geriatric Medicine, Chinese Academy of Medical Sciences, Beijing 100730, China; ^2^Peking University Fifth School of Clinical Medicine, Beijing 100730, China

## Abstract

With research progress on longevity, we have gradually recognized that cardiac aging causes changes in heart structure and function, including progressive myocardial remodeling, left ventricular hypertrophy, and decreases in systolic and diastolic function. Elucidating the regulatory mechanisms of cardiac aging is a great challenge for biologists and physicians worldwide. In this review, we discuss several key molecular mechanisms of cardiac aging and possible prevention and treatment methods developed in recent years. Insights into the process and mechanism of cardiac aging are necessary to protect against age-related diseases, extend lifespan, and reduce the increasing burden of cardiovascular disease in elderly individuals. We believe that research on cardiac aging is entering a new era of unique significance for the progress of clinical medicine and social welfare.

## 1. Introduction

According to “World Population Prospects 2019: Highlights,” elderly people above 65 years of age accounted for approximately 9.09% of the global population in 2019 and are expected to increase to 16.67% by 2050 as a result of declining fertility and mortality rates. In Japan, the most aging country in the world, the proportion of the population aged 65 and above has increased to 28.4%. In China, the proportion of people aged 65 and over in the total population has risen to 11.9%, an increase of 0.5% over the previous year. Some researchers predict that the average life expectancy of humans will increase four years by 2040 with improving health care and living conditions. The average life expectancy could exceed the age of 85 in some countries, such as Japan, Singapore, Spain, and Switzerland [1]. The aging of the population leads to a significant increase in the prevalence of age-associated diseases, especially cardiovascular disease (CVD) [2]. According to statistics, 20% of Americans will be over 65 years old, and nearly half of adults will suffer from CVD by 2030 [3]. CVD is the leading cause of death worldwide, and the largest independent risk factor is cardiac aging [4]. Therefore, it seems reasonable to focus on exploring the underlying mechanisms of cardiac aging and ameliorating or preventing the development of cardiac aging.

In 1939, McCay et al. showed that caloric restriction (CR) could increase the lifespan of rodents. The researchers demonstrated the plasticity of the aging process for the first time, which was a milestone in the field of aging research [5, 6]. Some studies on cardiac aging research showed that the hearts of young mice were composed of cardiomyocytes with different aging stages and functions. Traditionally, the heart was defined as an organ composed of a certain number of cardiomyocytes, which were no longer altered shortly after birth and maintained for life. This observation suggests the presence of senescent cardiomyocytes. Many senescent cardiomyocytes contribute to changes in cardiac structure and function, including impaired systolic reserves, diastolic dysfunction, and cardiac hypertrophy [7]. As the number of aging people continues to increase worldwide, it is critical to investigate changes in cardiac structure and function during cardiac aging. In this review, we will focus on the molecular mechanisms involved in cardiac aging and the possible interventions and treatments. Insights into the mechanisms of cardiac aging will be useful for reducing the incidence of CVD and providing guidance for safe and effective interventions and treatments for cardiac aging.

## 2. Mechanism of Cardiac Aging

Accumulating evidence suggests that the heart undergoes complex phenotypic changes during cardiac aging, including pathological myocardial remodeling, left ventricular systolic and diastolic dysfunction, cardiac hypertrophy, arrhythmia, microcirculatory dysfunction, and heart failure (HF) [8]. These biological changes can mediate a decline in cardiac function and increase heart vulnerability to stress, thus, significantly increasing the risk of CVD. As a result, the incidence of CVD, such as coronary heart disease, myocardial infarction (MI), stroke, and atherosclerosis, increases exponentially with age. Several mechanisms of cardiac aging have been proposed, such as oxidative stress, mitochondrial dysfunction, autophagy, telomere damage, noncoding RNAs, and aberrant mTOR (mechanical or mammalian target of rapamycin) signaling.

**Figure 1:**
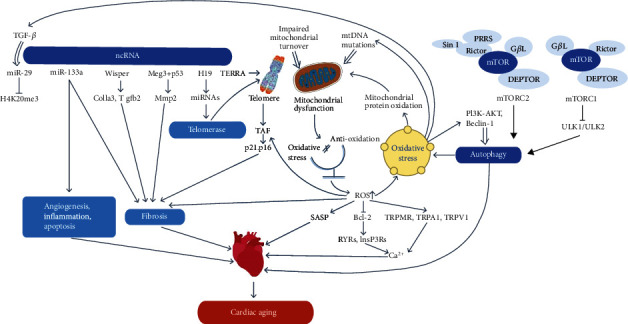
Key mechanisms of cardiac aging. Abnormal expression of ncRNAs can cause dysregulation of their downstream target genes, which cause telomere damage and cardiac aging. MtDNA mutations, mitochondrial protein oxidation, and impaired mitochondrial turnover can cause the loss of mitochondrial function and lead to inadequate ATP synthesis and increased ROS production. Increased ROS can lead to further mitochondrial dysfunction, forming a vicious cycle. Elevated ROS also lead to telomere dysfunction, DNA damage, and autophagy. Wisper indicates Wisp2 super-enhancer–associated RNA; Meg3: maternally expressed gene 3; TERRA: telomeric repeat-containing RNA; TAF: telomere-associated DNA damage foci; mtDNA: mitochondrial DNA; ROS: reactive oxygen species; and SASP: senescence-associated secretory phenotype.

Below, we introduce a few critical mechanisms associated with cardiac aging (see Figure 1).

### 2.1. Oxidative Stress

Free radicals are a class of atoms or groups with strong oxidizing traits, such as reactive oxygen species (ROS), which are highly unstable and easily react with other molecules. In 1956, Harman proposed the free radical theory of aging and suggested that endogenous free radicals might arise from oxidation-reduction reactions catalyzed by oxidase during fundamental metabolic processes, and aging-associated diseases are attributed to the long-term harmful effects of free radicals on cells and tissues to some extent [9]. Further studies have shown that disturbances in ROS under various stimuli are associated with the occurrence and development of multiple human diseases, such as inflammation [10], fibrosis [11], and tumorigenesis [12]. Cardiomyocytes require increased energy to maintain their functions. Compared with other tissues, the heart has higher basal oxygen consumption and produces more ROS [13]. Studies have shown that ROS are involved in the occurrence and development of multiple CVDs, such as hypertension, atherosclerosis, cardiac hypertrophy, and HF [14].

Indeed, some studies have indicated the important effects of oxidative stress on the development of cardiac aging [15]. A key discovery was that the overexpression of catalase targeted to mitochondria (mCAT) attenuated cardiac aging in mice. All age-related changes, such as the accumulation of oxidized mitochondrial proteins, increased mitochondrial DNA (mtDNA) mutations, and increased ventricular fibrosis, are significantly attenuated in mCAT-overexpressing mice [16]. Research has shown that TGF-*β*/Smad is one of the important pathways regulating injury-induced senescence and physiological senescence [17]. Oxidative stress can activate TGF-*β*, which in turn leads to acute accumulation of miR-29 and contributes to cardiac aging in vivo [18]. The blockade of TGF-*β* signaling can improve cardiac function in aging mice, which is highly beneficial to the intervention and treatment of cardiac aging. Moreover, ROS can enhance Ca^2+^ signal transduction by inhibiting Bcl-2 and increasing the sensitivity of ryanodine receptors (RyRs) and inositol 1,4,5-trisphosphate receptors (IP3Rs) [19]. ROS can also activate TRPM2, TRPA1, and TRPV1 channels, which control the release of Ca^2+^ [20]. Enhanced Ca^2+^ signal transduction leads to CVDs, such as cardiac hypertrophy and HF.

Studies have shown that enzymes associated with oxidative reactions are also involved in the occurrence of cardiac aging. For example, mice with mitochondrial superoxide dismutase (SOD) deficiency show seriously damaged neurons and cardiomyocytes and even exhibit dilated cardiomyopathy [21]. Consistently, monoamine oxidase (MAO) is also associated with oxidative stress. MAO is located on the outer mitochondrial membrane and is widely distributed in nerve tissue and the heart. Notably, MAO catalyzes the oxidative deamination of the substrate monoamine and produces hydrogen peroxide, participating in the production of intracellular ROS and playing a key role in the development of cardiac aging [22]. Depending on the substrate and the sensitivity of the inhibitor, MAOs are divided into two forms: isozyme A (MAO-A) and B (MAO-B) [23]. Overexpression of cardiomyocyte-specific MAO-A leads to elevated ROS in mice and induces cardiomyocyte necrosis and cardiac insufficiency [22]. These evidences suggest that oxidative stress is a key mediator of heart injury and cardiac aging, and it may be a clinical therapeutic target.

Because oxidative stress participates in the occurrence and development of cardiac aging, some researchers have suggested using antioxidants to prevent or even reverse cardiac aging, but increasing evidence shows that this intervention is not feasible. Antioxidant has been disappointing in many studies. And it is unclear whether the lifespan of certain species will increase under mild stress after supplementing with antioxidants. Low concentrations of ROS may play a protective role by triggering defense mechanisms against cell damage. The benefits induced by a low concentration of ROS can be explained by mitohormesis. Mitohormesis, a specific form of hormesis, means that high levels of free radicals are associated with cell injury and the inflammatory response, but a moderate increase in free radicals is related to a variety of signaling pathways, stimulating cells to enhance the protective mechanism to decrease injury [24]. Mitohormesis can induce a wide range of cytoprotective effects, adjust cellular metabolism, promote cell survival, and improve immunity via mitonuclear communication. Mitonuclear communication is an interaction mechanism between mitochondria and nuclei and includes a wide range of cytoplasmic and nuclear signaling pathways, such as ROS, the mitochondrial unfolded protein response (UPRmt), and mitochondrial metabolites [25, 26]. Contrary to the traditional view that ROS are a harmful metabolic byproduct, mitohormesis proves that a low concentration of ROS is essential for cell function and important for health. ROS can mediate vital signaling pathways that regulate cell survival and proliferation in physiological states and participate in mitosis. For example, some studies show that ROS play a key role in heart development. ROS lead to DNA damage and myocardial cell cycle arrest, which determines the transition of cardiomyocytes to postmitotic states. Consistent with this finding, other studies have shown that Prdx1 reduces cardiomyocyte apoptosis induced by myocardial ischemia-reperfusion injury through ROS-activated MAPK pathways [27].

### 2.2. Mitochondrial Dysfunction

Some cell biology studies have shown that cellular senescence, in particular cardiomyocyte senescence, is usually associated with functional organelles whose dysfunction leads to oxidative stress, the misfolding of proteins, and even cell death [28]. Particularly, there is increasing evidence that mitochondrial dysfunction is emerging as an important factor that can influence cardiac aging and is one of the common pathological features of cardiac aging. The mitochondrion, a versatile and semiautonomous organelle encapsulated by two layers of membranes in most eukaryotic cells, is the major site of aerobic respiration and the generation of energy (ATP) in cells and is called the power house [29]. MtDNA encodes two rRNAs, 22 tRNAs, and 13 peptides that participate in the synthesis of protein complexes in the electron transport chain in humans. MtDNA point mutations and deletions increase with age in various tissues in humans and rodents [30, 31]. More importantly, mtDNA mutations usually affect tissues and organs with high energy needs, such as the brain, muscle, and heart [32, 33]. Mitochondria are damaged or mtDNA is mutated by endogenous and external stimulation, resulting in an imbalance between oxidative stress and antioxidation and increased ROS production, thereby destroying the stability of the genome. More importantly, some studies have shown that an increase in ROS can also disrupt the tricarboxylic acid cycle and electron transfer chain, further exacerbating mitochondrial damage, which is called the vicious cycle theory of mitochondria [34].

It is indisputable that studies on oxidative stress also help us understand the relationship between mitochondrial and cardiac aging. According to their distribution, mitochondria in cardiomyocytes are divided into two types: interfibrillar mitochondria and subsarcolemmal mitochondria. Fusion and fission proteins are very sensitive to pathological changes in cardiomyocytes and can regulate mitochondrial biogenesis and morphological changes. In 1972, Harman identified that mitochondria play a key role in free radical production [35]. Indeed, numerous publications have demonstrated that mitochondria are major sources of intracellular ROS [36] and senescence-associated secretory phenotype (SASP), which are essential factors that drive cardiomyocyte senescence [37]. Notably, mitochondria produce superoxide, which is one of the most effective mediators that regulate cardiomyocyte senescence [35]. Given that mitochondria are the main organelles that produce energy by aerobic respiration, it is not surprising that mitochondrial dysfunction is directly associated with the development and progression of cardiac aging [38]. Numerous studies have demonstrated that the volume density of mitochondria is extremely high in cardiomyocytes [39]. The heart generally functions in a manner that is highly dependent on the energy supply from mitochondria. It was shown that more than 90% of the ATP consumed by cardiomyocytes comes from mitochondria. Therefore, the functional state of mitochondria directly affects cardiac function. Studies have shown that mitochondrial morphological abnormalities, mtDNA mutations, and mitochondrial unfolded protein increases are the main causes of cardiac aging [40].

Cardiomyocytes have the most abundant mitochondria in all cells, and mitochondrial dysfunction is also one of the most important characteristics of cardiac aging; therefore, the role of mitochondrial dysfunction in cardiac aging cannot be ignored. During cardiac aging, the increased demand for oxygen and energy of cardiomyocytes is caused by increased afterload and cardiomyocyte hypertrophy. However, due to impaired diastolic function, coronary perfusion is reduced in the left ventricle, resulting in insufficiency of oxygen and energy. Therefore, cardiomyocytes are more susceptible to mitochondrial dysfunction than any other cells. Mitochondrial ROS production increases gradually in cardiomyocytes during cardiac aging, accompanied by significantly weakened respiratory function and Ca^2+^ retention, which lead to cardiomyocyte injury and functional loss [41]. Mitochondrial dysfunction increases cardiac aging-related protein expression and apoptosis-inducing factor- (AIF-) associated cardiomyocyte apoptosis and exacerbates cardiac remodeling in aging mice [42]. However, further investigation is needed to determine the specific molecular mechanisms by which the senescence of cardiomyocytes is induced by mitochondrial dysfunction.

### 2.3. Impaired Autophagy

In the 1970s, Ashford and Porter identified a self-eating phenomenon in cells [43] that regulates homeostasis, which was named autophagy. Autophagy is a highly conserved and lysosomal-dependent biological process that is responsible for the degradation and recycling of long-lived or misfolded proteins and damaged organelles, thereby supporting cell metabolism and the renewal of organelles [44]. A key discovery was the demonstration that autophagy is triggered by class III phosphatidylinositol 3-kinase (PI-3K) and Beclin-1. There are three types of autophagy: microautophagy, chaperone-mediated autophagy (CMA), and macroautophagy [45]. Another key demonstration was the fact that autophagy is a double-edged sword in many species and tissues under physiological and pathological conditions. This finding is not surprising, as autophagy contributes to cell survival by removing damaged organelles or cellular components, but excessive autophagy also promotes cell death [46]. Therefore, autophagy is widely involved in the physiological and pathological processes of most living cells under different conditions, such as metabolism, longevity, growth and development, and a variety of diseases, including cardiac aging [47].

Autophagy generally clears misfolded proteins, damaged mitochondria, or DNA to maintain the structure and function of various cells and organs [48]. There are two main autophagy-related signaling pathways in cardiomyocytes: the MLC/FAK/AKT/mTOR-mediated inhibitory pathway and the Beclin1-mediated activation pathway. Autophagy is essential for maintaining cellular and protein homeostasis and plays a critical role in decreasing cardiac injury and sustaining cardiac function during cardiac aging [49]. However, decreases in autophagic activity and autophagic flow are found in the aging heart [50]. Impaired autophagy leads to the loss of cardiac tissue homeostasis and cardiac dysfunction with age, causing cardiac aging. For example, heart-specific knockdown of Atg5, an autophagy gene, reduces mitochondrial respiration and accelerates left ventricular hypertrophy and cardiac aging [51]. The inhibition of autophagy or autophagy gene mutation contributes to the occurrence of cardiac aging and neurodegenerative diseases [52]. Therefore, disruption of autophagy leads to cardiovascular abnormalities and cardiac dysfunction in mice.

Cardiac aging is characterized by myocardial hypertrophy, fibrosis, misfolded proteins, and mitochondrial dysfunction. Thus, autophagy and autophagic flux are usually reduced in cardiomyocytes during aging, whereas the accumulation of misfolded proteins and impaired organelles leads to cardiac dysfunction in mice that lose autophagy. Therefore, it has been shown that enhancing autophagy can improve cardiac function and alleviate pathological changes associated with cardiac aging by removing dysfunctional organelles and misfolded proteins. For example, inhibiting Akt/mTORC1 signaling can enhance autophagy, thereby inhibiting cardiac aging [53]. The deletion of Rho-associated coiled-coil-containing protein kinase (ROCK)1 and ROCK2 reduces cardiac fibrosis during cardiac aging by promoting age-related autophagy in mice [54]. In recent years, scientists have modified several genes associated with autophagy in some animal models to alleviate cardiac aging. In rodents, autophagy-related genetic modifications significantly reduce oxidative stress, improve myocardial contractile function, and resist cardiomyocyte senescence. Heat shock protein 27 (HSP27) can protect the heart from ischemia or damage from external stimuli. HSP27 overexpression inhibits the accumulation of LC3-II and p62 and attenuates cardiac aging-induced function damage in myocardial-specific transgenic HSP27 mice [55].

These studies show that the inhibition of autophagy contributes to cardiac aging, while enhancing autophagy has an antiaging effect on the heart. However, there is also evidence that an excessive increase in autophagy can also cause cardiac damage and accelerate cardiovascular aging. For example, palmitic acid-induced insulin resistance is accompanied by excessive autophagy and leads to the apoptosis of H9c2 cells [56]. Increasing evidence suggests that microRNAs (miRNAs) can also inhibit cardiac aging by regulating autophagy-related genes. The downregulation of miR-26b-5p, miR-204-5p, or miR-497-3p expression can enhance autophagy by targeting ULK1, LC3B, or Beclin1, respectively, and significantly promote exercise-induced myocardial hypertrophy in rats [57]. Many studies have demonstrated the importance of autophagy in cardiac aging, but it is unclear whether enhancing autophagy will be a good strategy for patients with cardiac aging.

### 2.4. Telomere Damage

Telomeres are short repeats of DNA at the end of linear chromosomes in eukaryotic cells. Together with telomere binding proteins, telomeres form a special cap structure that protects genes from degradation and maintains chromosomal integrity and genome stability [58]. Currently, telomere biology suggests that telomeres become shorter as cells divide, eventually destroying the cap structure and leading to a sustained DNA damage response (DDR) and cellular senescence [59]. In different types of cells, telomeres shorten at different rates, depending on the ability of the cell to proliferate. In turn, telomere shortening also affects cell division and senescence. Telomere shortening has been shown to be a major cause [60] and biomarker of aging [61].

Similarly, studies have identified telomere shortening as a risk factor for CVD [62]. Short telomeres are observed in elderly mice, in mice with dystrophic cardiomyopathy [63], and in patients with dilated cardiomyopathy [64]. A vital discovery was that telomerase deficiency induces severely short telomeres, leading to cardiac dysfunction and myocardial remodeling [65]. However, notably, one of the common hallmarks of cardiomyocytes is that these cells are postmitotic, and cardiomyocytes have little ability to proliferate throughout the life cycle of the organism. Thus, telomere shortening does not reflect the senescence of cardiomyocytes. The hypothesis of telomere shortening can explain the senescence of cells with the ability to divide, including hematopoietic cells. However, this hypothesis is not enough to explain the senescence of postmitotic tissues, such as the heart.

Telomeres are vulnerable to oxidative stress, as under the stimulation of ROS, the single-strand breaks occur in telomeres and accumulate in multiple cells with age [66, 67]. Dysfunctional telomeres can recruit a variety of DNA damage reactive proteins, activate the DDR, limit cell proliferation, and promote cell senescence [68]. This form of telomere damage is called telomere-associated DNA damage foci (TAF) [69]. These findings suggest that telomere damage caused by stress leads to telomere dysfunction and cell senescence. More importantly, Anderson et al. confirmed that telomere injury resulted in cardiomyocyte senescence and was independent of telomere length [70]. In cardiomyocytes, length-independent telomere injury activates p21^CIP^ and p16^INK4a^, a classic senescence-inducing pathway, driving myocardial fibrosis and hypertrophy. During the aging process, mitochondrial dysfunction leads to continuous DNA damage in the telomere region, causing cardiac aging in humans and mice independent of cell division or telomere length.

These studies show that telomere damage is also an important mediator of the pathophysiology of cardiac aging, promotes the progression of telomere biology related to cardiomyocyte senescence, and highlights the directions for future research to treat cardiac aging. For example, the specific molecular mechanisms of telomere damage that affect cardiac aging are unclear. Are these findings applicable in primates or humans? How can the findings be translated into the clinical treatment of cardiac aging? To solve these problems, further exploration is needed.

### 2.5. Noncoding RNAs

Noncoding RNAs (ncRNAs) are functional RNAs that do not encode proteins and include miRNAs, long noncoding RNAs (lncRNAs), small interfering RNAs (siRNAs), and small nucleolar RNAs (snoRNAs) [71]. Multiple studies suggest that the overexpression or inhibition of key ncRNAs is involved in various diseases, including CVD [72]. Further studies suggest that ncRNAs have powerful regulatory effects on cardiac aging under diverse pathophysiological states [73].

miRNAs are evolutionarily conserved ncRNAs containing 18-25 nucleotides and are negative posttranscriptional gene regulators that bind to the 3′-untranslated region of target genes. Emerging evidence has revealed that miRNAs play important roles in regulating cardiac aging [74]. For example, miR-34a expression is increased in aging hearts. The inhibition of miR-34a attenuates myocardial infarction- (MI-) induced myocardial fibrosis improves capillary density in MI border zones and alleviates cardiac dysfunction in mice. The overexpression of phosphatase 1 nuclear targeting subunit (PNUTS, also known as PPP1R10), a target gene of miR-34a, reduces DNA damage and cardiomyocyte apoptosis and improves cardiac function [75]. miR-125b attenuates Ang II-induced cardiac fibrosis by silencing antifibrotic factors [76]. miR-378 also inhibits cardiac fibrosis by targeting MKK6 and inhibiting p38 signaling [77]. Lyu and colleagues discovered that TGF-*β* triggered cellular senescence and cardiac aging through the loss of H4K20me3 induced by miR-29 in aged mice [18]. TGF-*β*/Smad is one of the primary pathways that regulate pathological and physiological aging [17]. miR-133a is one of the most abundant miRNAs in the heart. Studies have shown that miR-133a inhibits angiogenesis, inflammatory responses, cardiomyocyte apoptosis, and fibrosis and is involved in early pathological changes in ischemic myocardial disease and subsequent cardiac remodeling [78]. Hence, miRNAs may be a therapeutic target for cardiac aging.

lncRNAs, defined as ncRNAs with lengths of more than 200 nucleotides, play important roles in many cellular processes by regulating gene expression at the transcriptional or posttranscriptional level. Studies demonstrate that there are three main mechanisms of action for lncRNAs: most lncRNAs bind to ribonucleoprotein (RNP) and regulate its function, lncRNAs act as miRNA sponges, and some lncRNAs target DNA and recruit chromatin-modified proteins to form RNA–DNA–protein complexes.

Studies on lncRNAs have further revealed the effects of ncRNAs on cardiac aging [79]. For example, telomeric repeat-containing RNA (TERRA) has been shown to regulate telomere structure, induce telomere RNA damage responses, and limit cell proliferation [80]. Similarly, lncRNA H19 is a precursor of multiple miRNAs associated with high levels of telomerase expression and is involved in inhibiting cell proliferation [81] and regulating cell senescence [82]. Studies have shown that lncRNA Wisp2 super-enhancer–associated RNA (Wisper) is highly expressed in cardiac fibroblasts. This finding was followed by the discovery that the upregulation of Wisper promotes the expression of profibrotic genes, including Colla3 and Tgfb2, which in turn induces cardiac fibrosis and left ventricular dysfunction [83]. Studies have shown that maternally expressed gene 3 (Meg3) binds p53 and induces the expression of Mmp2 promoters, thereby promoting cardiac fibrosis and participating in subsequent cardiac remodeling and HF with preserved ejection fraction (HFpEF) [84]. Cardiac autophagy inhibitory factor (CAIF) regulates myocardial transcription, inhibits cardiac autophagy, and alleviates MI by directly binding to p53 [85]. However, there have been few detailed reports on the effects of lncRNA on cardiac aging, and the possibility of lncRNA becoming a biomarker of cardiac aging and a target for therapeutic interventions remains to be further investigated.

### 2.6. Dysregulation of mTOR Signaling

Target of rapamycin (TOR) proteins, an evolutionarily conserved and atypical serine/threonine kinase, was first discovered through rapamycin research. TOR has many biological functions and plays an important role in regulating cell metabolism, survival, growth, proliferation, energy level, and homeostasis. Genetic and pharmacological modulations of TOR prolong lifespan in many organisms [86, 87]. TOR is involved in the signal transduction of diverse processes regulating cardiac aging, including gene transcription, lipid and protein synthesis, oxidative stress, autophagy, and mitochondrial function. The TOR gene in mammals is called the mechanical (or mammalian) target of rapamycin (mTOR). mTOR exists in two protein complexes with different structures and functions. The first is mTOR complex 1 (mTORC1), which is composed of mTOR, Raptor, G*β*l, and DEPTOR and is inhibited by rapamycin. The second complex is mTOR complex 2 (mTORC2), which is made up of mTOR, Rictor, *β*l, Sin1, PRR5/Protor-1, and DEPTOR and is insensitive to short-term rapamycin treatment [88]. There is growing evidence that mTOR signaling is activated in many diseases, including cancer [89], neurodegenerative disorders [90], obesity [91], chronic obstructive pulmonary disease [91, 92], pulse arterial hypertension [93], and CVD [53].

The mTOR signaling pathway is a key regulator of protein synthesis and cell metabolism and is located in the endoplasmic reticulum and ribosomes. Older mice show higher mTOR activity in the heart than younger mice. Several studies have also elucidated the relationship between mTOR and cardiac aging. Current research has shown that autophagy is suppressed as mTOR activity increases, which can lead to cardiac fibrosis [54]. The NLR family pyrin domain containing 3 protein (NLRP3) inflammasome plays a central role in cardiovascular events related to aging. Studies have shown that the deletion of NLRP3 inhibits the mTOR pathway and induces autophagy in mice. Marin-Aguilar et al. showed that compared with old wild-type male mice, old NLRP3^−/−^ male mice showed improved autophagy and cardiac aging and extended lifespans by suppressing the PI3K/AKT/mTOR pathways [94]. mTOR can also drive cardiovascular dysfunction and is involved in atherosclerosis [95, 96]. mTOR inhibition triggers autophagy, promotes cell survival and cholesterol efflux, and inhibits inflammation and plaque rupture. For example, lovastatin prevents intimal hyperplasia by inhibiting mTOR in smooth muscle cells [97].

mTORC1 is a major regulator of autophagy and can inhibit autophagosome formation by increasing the phosphorylation of the serine/threonine protein kinase Atg1 (ULK1/ULK2 in mammals) [98]. As a key autophagy regulator, mTORC1 plays an important role in regulating cardiac function, cardiomyocyte survival, and cardiac tissue homeostasis. mTORC1 can promote age-related decline in cardiac function in Drosophila [99] and high-fat diet-induced cardiomyopathy [100]. Heart-specific mTORC1 overexpression increases stress-induced HF [101]. In some experimental models, mTORC1 inhibition can promote cardiomyocyte survival, thereby reducing cardiac aging and prolonging biological life. This may be associated with the regulation of autophagy, oxidative stress, and inflammation. In MI-induced chronic HF rat models, mTORC1 inhibition promoted autophagy, reduced cardiomyocyte apoptosis, improved cardiac function, and inhibited cardiac remodeling [53]. A recent study also showed that inhibition of mTORC1 could continuously trigger autophagy, improve cardiac function, and inhibit cardiac hypertrophy and cardiac aging in aged female and male mice [102]. Rapamycin, an mTORC1 inhibitor, mediates mTORC1 inhibition, induces autophagy, and protects cardiomyocytes from oxidative stress in mice [103].

In contrast to mTORC1, mTORC2 is a positive regulator of autophagy. The results of Chang et al. show that mTORC2 can regulate autophagy and heart health during cardiac aging. Heart-specific knockdown of TGF*β*-INHB/activin-like protein DAW, a novel upstream regulator of mTORC2, activates mTORC2 signaling, induces autophagy, and alleviates age-related cardiac dysfunction, including arrhythmia and bradycardia [104]. MTORC2 can also promote cell survival by activating Akt signaling, regulate cytoskeletal dynamics and glucose homeostasis by activating PKCa, and reorganize the actin cytoskeleton. Research shows that the disruption of the RPTOR-independent companion of MTOR complex 2 (RICTOR), a subunit of mTORC2, can also cause cardiac dysfunction in mice [105].

In addition to these mechanisms, other pathogenic factors that contribute to cardiac aging include apoptosis, genetic and epigenetic modifications, metabolic dysregulation, the secretion of inflammatory factors, and changes in iron status [7, 106, 107].

## 3. Possible Treatment Strategies for Cardiac Aging

### 3.1. Caloric Restriction

Caloric restriction (CR) refers to reducing caloric intake without malnutrition. CR is a repeatable dietary intervention and is currently known to improve cardiac aging and prolong lifespan in various organisms and humans [108]. In rodent models of cardiac aging, CR attenuates cardiac hypertrophy and fibrosis and alleviates cardiac dysfunction [109]. Preclinical and clinical evidences also suggest that CR is an effective way to improve cardiac remodeling and inhibit cardiac aging [110].

One of the underlying mechanisms by which CR protects against cardiac aging is the induction of autophagy. CR regulates autophagy through multiple upstream regulators, such as mTOR, Sirt1, and AMPK [111, 112]. MTOR signaling is the most well-known regulator of autophagy, cell homeostasis, and longevity. CR can inhibit the mTOR pathway, thereby inducing autophagy, regulating cardiac metabolism, and attenuating age-dependent cardiac hypertrophy and diastolic dysfunction [108]. CR can also activate the PI3K/Akt pathway to partially reduce aging-induced cardiac insulin resistance, enhance myocardial contractility, and prevent cardiac aging [113].

One of the key mechanisms of cardiac aging is the increased production of ROS, which damages proteins, lipids, and DNA and induces oxidative stress in cardiomyocytes. As mentioned previously, there is evidence that slowing the formation of macromolecules in oxidatively damaged cells may improve cardiac function and prevent cardiac aging. Studies have shown that CR exerts cardioprotective effects partly by improving redox homeostasis and inhibiting oxidative stress in aging male rats, even when body weight remains stable [114]. In this context, CR has the potential to improve cardiac aging.

In addition, CR also affects many risk factors for cardiac aging in laboratory animals and humans. In rats, CR can reduce cardiomyocyte apoptosis and attenuate cardiac remodeling and fibrosis [115]. CR can also reduce myocardial hypertrophy and prevent cardiac aging by improving metabolism and lowering blood pressure [116]. According to reports, CR can reduce proinflammatory cytokines, increase anti-inflammatory cytokines, and reduce the inflammatory response during cardiac aging [117]. CR inhibits aging-related inflammation, improves arterial function, and reduces the incidence and mortality of CVD in rats [118]. It is well known that many diseases, such as diabetes and obesity, can damage the structure and function of the heart and lead to cardiac aging [91], while CR can inhibit oxidative stress and improve cardiac remodeling and cardiac function [119]. Nicklas et al. showed that aerobic exercise combined with CR can improve cardiorespiratory health and control blood glucose more effectively than individual exercise [120].

### 3.2. Cell Therapy

Mechanisms of cell therapy include (1) generating new cardiomyocytes, (2) secreting paracrine factors, (3) reducing myocardial fibrosis, and (4) enhancing myocardial contractility. The results of preclinical and clinical studies have shown that cell therapy is a good strategy for repairing and regenerating damaged hearts. Multiple types of cells have the potential to treat cardiac aging, including mesenchymal stem cells, peripheral blood monocytes, cardiac progenitor cells, and pluripotent stem cells [121–124]. At the beginning of this century, adult stem cells, such as bone marrow-derived stem cells [125], have shown multiple benefits in clinical testing, such as reducing scar tissue and improving cardiac output, which demonstrates that cell therapy is potentially translatable to treat cardiac aging in humans [126]. These findings also led to the rapid development of cell therapy.

However, it is still controversial whether cell therapy is safe and efficacious for preventing and treating cardiac aging [127]. Cardiosphere-derived cells (CDCs) are cardiac progenitor cells that can be obtained by endomyocardial biopsy [128, 129] and differentiate into three major cell types in the heart: cardiomyocytes, endothelial cells, and smooth muscle cells [130]. Previous studies have shown that transplantation of CDCs into the aging hearts of rats enhances cardiac function, exercise ability, and hair regeneration [131]. However, Zhao et al. found that CDCs do not improve cardiac function and systemic function in aging mice, in contrast to the control group [132]. In addition, there are some problems with cell therapy, such as the low efficacy of treatment, the selection of cell types, and the limitations of using cells as therapeutic products [133].

### 3.3. Rapamycin

Rapamycin, the first and only macrolide drug approved by the US Food and Drug Administration (FDA), can directly inhibit mTORC1. Rapamycin was first used as an antifungal drug [134]. In 1977, researchers found that rapamycin has immunosuppressive effects. In 1999, the FDA approved rapamycin for preventing or ameliorating immune rejection in kidney transplantation. Studies have shown that rapamycin also reduces the incidence of many diseases, such as cerebral malaria [135], pancreatic cancer [136], and diseases of the blood system [137]. The FDA approved rapamycin for the treatment of lymphangioleiomyomatosis (LAM) in May 2015, and it was also the first drug approved for treating such rare diseases. Currently, rapamycin is widely accepted as the compound with the greatest impact on lifespan [138]. Studies have shown that rapamycin can inhibit cell senescence and prolong the lifespan by targeting conserved aging pathways in many organisms, such as nematodes, rodents, and primates.

Diastolic dysfunction is a common problem in cardiac aging, but there is currently no targeted treatment. Rapamycin has been shown to reverse age-related diastolic dysfunction in rodents [139] and dogs [140]. In older mice, rapamycin can continue to improve diastolic function and myocardial stiffness, even after stopping treatment [102]. Dai et al. also suggested that short-term treatment with rapamycin could improve or even reverse the decline in diastolic function and left ventricular hypertrophy during cardiac aging [141].

Rapamycin can also activate autophagy by directly inhibiting mTORC1 in the heart. For example, rapamycin reverses age-associated oxidative stress as well as cardiac and vascular dysfunction by activating AMPK pathways, inhibiting mTOR pathways, inducing autophagy, and promoting mitochondrial biogenesis [142, 143]. Rapamycin can enhance autophagy, promote cardiomyocyte survival, and delay autophagy-related 5 (Atg5) siRNA-induced cardiac aging by inhibiting the Akt/mTORC1 pathway in mice [144].

Moreover, rapamycin can inhibit cardiac aging by regulating oxidative stress, inflammation, and organelle function. Studies have shown that rapamycin can reduce mitochondrial ROS and inhibit cardiac hypertrophy and cardiac aging by inhibiting mTORC1 [145, 146]. Das et al. demonstrated that rapamycin could reverse age-related metabolic changes, thereby exerting a cardioprotective effect in experimental models [147]. However, rapamycin also has the disadvantages of a high frequency of administration and side effects, such as anemia and acute nephrotoxicity [148].

The treatment of cardiac aging has become a rapidly growing healthcare burden worldwide. However, the continuous increase in our understanding of the pathogenesis of cardiac aging provides new pathways to protect cardiac structure and function during the process of aging. Some studies in animal models suggested that other drugs could also maintain cardiac structure and function and protect against cardiac aging. Metformin, an AMPK activator, has been shown to reduce myocardial contractile dysfunction during cardiac aging [149]. Some studies have shown that nicotinamide derivatives, such as the CD38 inhibitor 78c, have the potential to serve as clinical drugs for cardiac aging. Experimental results show that nicotinamide derivatives stimulate autophagy and mitochondrial phagocytosis, increase the level of NAD^+^, and improve cardiac function in humans and rodents [150, 151]. Second, spermidine, a natural polyamine, can induce autophagy and inhibit histone acetyltransferase, inflammation, and oxidative stress, thereby enhancing diastolic function, attenuating cardiac hypertrophy and remodeling, and delaying the development of HF [152]. Agelastatin A, a novel inhibitor of osteopontin (OPN), can treat cardiac aging and induce fibroblast senescence in mice [153].

As mentioned above, these drugs show great potential against cardiac aging in animal models. However, it is still unclear whether these drugs can clinically treat cardiac aging in humans. A key question is how to assess the therapeutic effect of these drugs in clinical trials. Moreover, whether these interventions can prevent or ameliorate other age-related diseases or reduce the symptoms of patients with multiple disorders needs further evaluation. It is critical to analyze the potential risk of drug side effects on other diseases. To address these problems, further exploration is necessary. However, experimental data from preclinical models suggested that it is still possible to prevent and treat cardiac aging.

## 4. Conclusions

**Figure 2:**
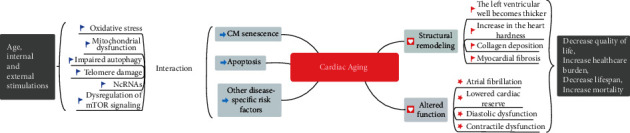
The concept of cardiac aging and its biological mechanism. Cardiac aging is a pathophysiological process affected by many factors triggered under various intrinsic and external stimuli with age. Many factors can affect key cellular processes and pathways during cardiac aging, such as oxidative stress, mitochondrial dysfunction, impaired autophagy, telomere damage, ncRNAs, and dysregulation of mTOR signaling. These factors can also influence each other and lead to cardiomyocyte senescence, apoptosis, and other cell damages. Cardiac aging causes changes in heart structure and function, such as progressive myocardial remodeling, decreases in systolic and diastolic function. CM indicates cardiomyocytes.

Aging is characterized by the loss of cell renewal and repair capacity, which leads to increased morbidity and mortality in multiple age-associated diseases, including cardiac aging, and an increasing healthcare burden around the world. Under the aging trend of the global population, basic research and clinical treatment of these diseases have become more important. Cardiac aging is a rapidly developing research field and has received extensive attention. Several lines of evidence support that cardiac aging is a pathophysiological process affected by many factors triggered by endogenous and exogenous stimulating factors and is coregulated by multiple senescence-associated signaling networks (see Figure 2). Because of the increase in morbidity and mortality, there is a strong need to find effective therapeutic strategies to intervene or treat cardiac aging. Improved understanding of the underlying mechanisms of cardiac aging contributes to treating cardiac aging and reducing the risk of CVD.

## Data Availability

All data needed to evaluate the conclusions in the paper are present in the paper. Additional data related to this paper may be requested from the authors.
